# Strain Classification of *Mycobacterium tuberculosis* Isolates in Brazil Based on Genotypes Obtained by Spoligotyping, Mycobacterial Interspersed Repetitive Unit Typing and the Presence of Large Sequence and Single Nucleotide Polymorphism

**DOI:** 10.1371/journal.pone.0107747

**Published:** 2014-10-14

**Authors:** Sidra E. G. Vasconcellos, Chyntia Carolina Acosta, Lia Lima Gomes, Emilyn Costa Conceição, Karla Valéria Lima, Marcelo Ivens de Araujo, Maria de Lourdes Leite, Flávio Tannure, Paulo Cesar de Souza Caldas, Harrison M. Gomes, Adalberto Rezende Santos, Michel K. Gomgnimbou, Christophe Sola, David Couvin, Nalin Rastogi, Neio Boechat, Philip Noel Suffys

**Affiliations:** 1 Laboratory of Molecular Biology Applied to Mycobacteria, Oswaldo Cruz Institute, FIOCRUZ, Rio de Janeiro, Rio de Janeiro, Brazil; 2 Laboratory of Cellular Microbiology, Oswaldo Cruz Institute, FIOCRUZ, Rio de Janeiro, Rio de Janeiro, Brazil; 3 Instituto Evandro Chagas, Section of Bacteriology and Mycology, Belém, Pará, Brazil; 4 Hospital Municipal Rafael de Paula Souza, Municipal Secretary of Health, Rio de Janeiro, Rio de Janeiro, Brazil; 5 Centro de Referência Professor Hélio Fraga, Escola Nacional de Saúde Publica Sergio Arouca, FIOCRUZ, Rio de Janeiro, Rio de Janeiro, Brazil; 6 CNRS–Université Paris–Sud, Institut de Génétique et Microbiologie–Infection Genetics Emerging Pathogens Evolution Team, Orsay, France; 7 Supranational TB Reference Laboratory, Unité de la Tuberculose et des Mycobactéries, Institut Pasteur de Guadeloupe, Abymes, Guadeloupe, France; 8 Multidisciplinary Research Laboratory, University Hospital Clementino Fraga Filho – HUCFF, Federal University of Rio de Janeiro, Rio de Janeiro, Rio de Janeiro, Brazil; 9 Graduate Program in Clinical Medicine, Faculty of Medicine, University Hospital Clementino Fraga Filho, Rio de Janeiro, Rio de Janeiro, Brazil; St. Petersburg Pasteur Institute, Russian Federation

## Abstract

Rio de Janeiro is endemic for tuberculosis (TB) and presents the second largest prevalence of the disease in Brazil. Here, we present the bacterial population structure of 218 isolates of *Mycobacterium tuberculosis*, derived from 186 patients that were diagnosed between January 2008 and December 2009. Genotypes were generated by means of spoligotyping, 24 MIRU-VNTR typing and presence of fbpC^103^, RD^Rio^ and RD174. The results confirmed earlier data that predominant genotypes in Rio de Janeiro are those of the Euro American Lineages (99%). However, we observed differences between the classification by spoligotyping when comparing to that of 24 MIRU-VNTR typing, being respectively 43.6% vs. 62.4% of LAM, 34.9% vs. 9.6% of T and 18.3% vs. 21.5% of Haarlem. Among isolates classified as LAM by MIRU typing, 28.0% did not present the characteristic spoligotype profile with absence of spacers 21 to 24 and 32 to 36 and we designated these conveniently as “LAM-like”, 79.3% of these presenting the LAM-specific SNP *fbpC^103^*. The frequency of RD^Rio^ and RD174 in the LAM strains, as defined both by spoligotyping and 24 MIRU-VNTR loci, were respectively 11% and 15.4%, demonstrating that RD174 is not always a marker for LAM/RD^Rio^ strains. We conclude that, although spoligotyping alone is a tool for classification of strains of the Euro-American lineage, when combined with MIRU-VNTRs, SNPs and RD typing, it leads to a much better understanding of the bacterial population structure and phylogenetic relationships among strains of *M. tuberculosis* in regions with high incidence of TB.

## Introduction

Tuberculosis (TB) is an infectious disease with an effective treatment but remains an important cause of morbidity and mortality in many countries. In Brazil, 73.000 new TB cases and 4.600 cases that deceased were registered in 2011. The southeast region has 44.8% of all cases reported in the country and the state of Rio de Janeiro has the highest disease incidence (72.3/100,000) and mortality rate (5.7/100,000) in the country [1].

A couple of years ago, *Mycobacterium tuberculosis* (Mtb) strains designated as RD^Rio^ were reported as being a predominant genotype in Rio de Janeiro [2]. These strains have a deletion of 26.3 kb and seem to be restricted to the Latin American-Mediterranean (LAM) family. The RD^Rio^ strains have been associated with higher levels of recent transmission and of Multi-Drug Resistance (MDR) but there are contradictory data about their relation with disease severity [3], [4], [5], [6]. Another Region of Difference called RD174 has been described as a co-marker of RD^Rio^ and as a marker for the LAM type [7], [8], [9], [10] and strains with the RD174 deletion were found to have an increased secondary case rate ratio in San Francisco [10].

The most commonly used genotyping methods to characterize *M. tuberculosis* Complex (MTBC) isolates are IS*6110*-RFLP, 24-locus MIRU-VNTR, spoligotyping, detection of Single Nucleotide Polymorphisms (SNPs) and Large Sequence Polymorphisms (LSP). Typing of MTBC by IS*6110*-RFLP was considered the gold-standard during more than a decade [11], but is labor intensive. Currently, the two most commonly used methods are spoligotyping and Mycobacterium Interspersed Repetitive Units – Variable Number of Tandem Repeats (MIRU-VNTR) typing, based on the variability of the direct repeat (DR) locus [12] and of minisatellites [13], [14], respectively. However, for epidemiologic studies, spoligotyping overestimates the proportion of clustered strains and should be used in combination with MIRU-VNTR typing [15]. Spoligotyping alone is also unable to reveal exact phylogenetic relationships between MTBC strains, particularly for the classification of Euro-American strains, part of the PGG2/PGG3 group [9], [16], [17], [18]. These phylogenetic subtypes include the genotype families LAM, H, T, S and X, frequently observed in Brazil, in South America and elsewhere [2], [4]. [8], [18], [19], [20], [21], [22], [23].

The generalized use of spoligotyping and MIRU-VNTR resulted in the construction of large international databases of genotypes, allowing the study of global distribution and phylogenetic analysis of the distribution of *M. tuberculosis* worldwide [23], [24], [25], [26], [27], [28], [29]. Combined MIRU-VNTR typing and spoligotyping also helps in revealing epidemiologically meaningful clonal diversity of *M. tuberculosis* strain lineages and is useful to explore internal phylogenetic ramifications [8]. In addition, SNPs and LSPs represent robust markers for inferring phylogenies and for strain classification [30], [31], [32], [33], [34], [35]. Besides the presence of RDs, that of determinate SNPs is also a characteristic of LAM strains, [8], [9], [10] such as *fbpC^103^* in codon 103 (G to A) of the gene encoding antigen 85 Complex Ag85^c^ (Rv0129c) resulting in a Glu103Asp amino acid replacement in a protein that is involved in biosynthesis of cell wall components of *M. tuberculosis* [7], [36]. Other LAM-specific SNPs are (i) the silent C to G *ligB* mutation at genome position 3426795 [37], validated by the Abadia *et al.* [17] and (ii) the SNP at 240 codon of *mgtC* (C to T) that was discovered by Homolka *et al.* [38].

The objective of this study was to evaluate 24 MIRU-VNTR typing for alternative classification of strain families/lineages by means of phylogenetic tree building and comparison with MIRU databases (MIRU-VNTRPlus), as compared with spoligotypes-based classification (SITVITWEB and SITVIT2), and increase the consistency of the analysis by including a SNP and two LSPs. In addition, the differentiating power of both techniques in a population that is almost exclusively of the Euro-American lineages.

## Materials and Methods

### Study setting

The study was based on a convenience sampling of TB patients diagnosed between January 2008 and December 2009 at the “Hospital Municipal Raphael de Paula e Sousa”, Curicica, Rio de Janeiro, Brazil. Two hundred eighteen clinical isolates were obtained from 188 patients and 28 cases had more than one isolate, including 25 patients with two and three patients with three isolates. Multiple isolates from the same patient were included to evaluate the consistency of the results of genotyping and to verify eventual multiple infections.

### Ethics statement

This study was approved by the Research Ethics Committee of the Municipal Health and Civil Defense of the city of Rio de Janeiro Number 160/09 CAAE: 0182.0.314.000-9. Isolates of this study were obtained through bacteriological culture from clinical specimens of patients diagnosed at the Hospital Raphael and Paula Souza as part of routine diagnosis and drug susceptibility testing. The ethical committee stated that, as genotyping of *M. tuberculosis* isolates is complementary to routine diagnosis, there was no need for a written or verbal consent. No information other than that provided for the diagnosis was used.

### Culture, DNA extraction and identification of Mycobacterium tuberculosis complex

Sputum samples were cultured on Löwenstein-Jensen (LJ) medium following standard microbiological laboratory procedures as a routine of the Clinical Analysis Laboratory of “Hospital Raphael de Paula e Souza”. After cultivation, bacterial mass was re-suspended in 400 µl of sterile distilled water and heat inactivated at 90°C for one hour, followed by DNA extraction and purification using the CTAB method [39].

### Spoligotyping

Spoligotyping was performed either as described by Kamerbeek *et al.* (1997) [12], using commercially available kit from Ocimum Biosolutions (Hyderabad, India) according to the manufacturer's instructions or using microbead-based hybridization assay as described by Zhang *et al.* (2009) [40].

### MIRU-VNTR typing

Amplification of 24 MIRU-VNTR *loci* was performed by using a commercial typing kit (Genoscreen, Lille, France) and automated MIRU-VNTR analysis performed as previously described [14]. Fragment size of the amplicons was analyzed on a ABI 3730 DNA sequence analyzer (Applied Biosystems, California, USA) and number of copies of each locus was determined by automated assignment using the Genemapper 4.0 software (Applied Biosystems, California, USA). In the case of doubtful results, the size of the repeats was double checked by size estimation as compared to a DNA ladder (50 and 100 bp) and the positive control (H37Rv) on agarose gels and by comparing to a reference table as described [13].

### PCR-RFLP of *fbpC^103^*

For characterization of SNP *fbpC^103^*, we adapted the procedure described by Gibson *et al.* in 2008 [7]. Amplifications were performed in 50 µl reactions containing 40 pmol each of the primers Ag85C103F (5-CTG GCT GTT GCC CTG ATA CTG CGA GGG CCA-3) and Ag85C103R (5-CGA GCA GCT TCT GCG GCC ACA ACG TT-3), 2 mM MgCl2, 0.2 mM dNTPs, 1 U Taq DNA polymerase (Invitrogen, Brazil), buffer (10 mM Tris-HCl, 1, 5 mM MgCl2, 50 mM KCl, pH 8.3), 5% DMSO (v/v) and 10 ng of target DNA. Amplification was performed in a Vieriti Thermal Cycler (Applied Biosystems, Foster City, CA), starting with denaturation at 95°C for 5 min, followed by 45 cycles of 1 min at 95°C, 1 min at 60°C, 4 min at 72°C and final extension for 10 min at 72°C. The amplified products of 519 bp were analyzed on 2% agarose gels after staining with ethidium bromide. Fifteen microliters of the amplified products were subjected to enzymatic digestion with 1 U of *Mnl*I (New England BioLabs Inc. USA) at 37°C for four hours, generating three fragments of 365 bp, 96 bp and 48 bp for the wild type allele and two bands of 461 bp and 48 bp when the SNP G103A is present. Bands were detected in 3% agarose gel and their size estimated by comparison with a 100 bp DNA ladder (Invitrogen).

### RD174 deletion

For detection of RD174, we used the protocol described earlier [7], performing multiplex-PCR using two primers that anneal to the RD174 flanking regions and one to the internal sequence. For amplification, we used 40 pmol of each of the primers RD174F (5′-AGC TGC TCC GGC CGG TCG TCG TCC TTG TC-′3), RD174Fi (5′-TAT GCC GCA GCC CGG GCA TCC GTG ATT A-′3) and RD174R (5′-ATC GTG AAC GCA GCG GTT TCG ACG GCA TCT-′3) in a 50 µl reaction containing 2 mM MgCl2, 0.2 mM dNTP, 1 U Taq DNA polymerase, 1× buffer, 1M of betaine and 10 ng of bacterial DNA. Amplification was performed starting with 5 min incubation at 95°C, followed by 45 cycles of 1 min at 95°C, 1 min at 60°C, 4 min at 72°C and final extension for 10 min at 72°C. To determine the size of the amplicons, 10 µl of PCR product was applied in 2% agarose gel and after electrophoresis, bands of either 300 bp (intact RD174) or 500 bp (deleted) are observed.

### RD^Rio^ deletion

Detection of RD^Rio^ was performed using the multiplex PCR-protocol described by Lazzarini *et al.* (2007) [2]. For amplification, we used 20 pmol of each of the primers BridgeF (5′-CAC TCC GGC TGC CAA TCT CGT C′3), BridgeR (5′-CAC GAG GCT CGC GAA TGA GAC C-′3), IS1561F (5′-GAC CTG ACG GCC ACA CTG C′-3) and IS1561R (5′-CAC CTA CAC CGC TTC CTG CC′-3) in 50 µl reactions containing 2 mM MgCl_2_, 0.2 mM dNTP, 1 U Taq DNA polymerase), 1× buffer, 5% DMSO (v/v) and 10 ng of target DNA. Amplifications were performed starting with incubation for 5 min at 95°C, followed by 45 cycles of 1 min at 95°C, 1 min at 60°C, 4 min at 72°C and final extension of 10 min at 72°C. The amplified products of 1175 bp or 530 bp in the presence or absence of the deletion, respectively were analyzed in 1.5% agarose.

### Classification and definition of genotype-based lineages and families

Spoligotype and MIRU patterns were defined according to the definitions in the SITVITWEB database [23], (http://www.pasteurguadeloupe.fr:8081/SITVIT_ONLINE/) in the format of November 20, 2012. In addition, spoligotypes were compared with those in SITVIT2 (proprietary database of the “Institut Pasteur de la Guadeloupe”, which is an updated in-house version of the publicly released SpolDB4 and SITVITWEB [23], [24] to be released in 2014. This comparison led to the characterization of families and lineages by spoligotyping and Spoligotype International Types (SIT), MIRU International Types (12-MIT, 15-MIT AND 24-MIT) and 5 *loci* VNTR International Types (VIT). The SIT, MIT and VIT were designated identical patterns when shared by two or more patient isolates, whereas “orphan” patterns were those observed in a single isolate that did not correspond to any of the patterns present in the SITVIT2 database. In order to classify “orphan” patterns, we used SpotClust (http://tbinsight.cs.rpi.edu/run_spotclust.html).

The 24 MIRU-VNTR profiles and spoligotypes were also compared with the MIRU-VNTR*plus* database [28] available at (http://www.miru-vntrplus.org/MIRU/index.faces). The definition of lineages was done on 24 MIRU-VNTR loci by Best-match analysis and Tree-based identification using the categorical index. The Multi-locus Variable Number Tandem Repeat analysis (MLVA) MtbC 15 and MtbC 12 were determined.

### Data analysis

The number and size of genotype clusters were defined initially by introducing numerical values into an Excel table and different criteria for definition of clusters were used, being either individual identical spoligotypes or 15–24 MIRU-based genotypes, or by combining with Spoligotyping both. Excel tables were introduced into BioNumerics software (version 6.1, Applied Maths, Belgium) for construction of similarity matrices and phylogenetic trees, using the Jaccard Index (spoligotypes) and Categorical value (MIRU-VNTR) for construction of the Neighbor Joining (NJ) and Minimal Spanning Tree (MST).

The MIRU-VNTR allelic diversity (*h*) at each of the 24 loci was calculated by the equation described by Graur and Li [41] and the Diversity index was calculated as the Hunter Gaston diversity index (HGDI) [42]. Statistical analysis were performed using chi-square analysis with a confidence interval of 95% using Epi Info.Version 3.51 (Centers for Disease Control and Prevention, Atlanta, GA,USA), by using χ2 test or Fisher exact test for the comparison of proportions. A *p* value<0.05 was considered significant.

To visualize the classification difference observed between Spoligotyping and 24 loci MIRU-VNTRs, we constructed a Confusion Matrix for the frequency of correct and incorrect predictions. From the confusion matrix, the Positive Predictive Value or precision (PPV = TP/TP+FN), Accuracy rate (AAC = TP+TN/TP+FP+TN+FN), True Positive Rate or Sensitivity (TPR = TP/TP+TN), True Negative Rate or Specify (TNR = TN/TP+TN), Negative Predictive Value (NPV = TN/FN+TN) and Error Rate (ER = FP+FN/TP+FP+TN+FN) were calculated. (TP = True Positive, FP = False Positive, TN = True Negative, FN = False Negative).

## Results

### Classification by spoligotyping

The identification of spoligotype profiles as well as their definition to the family and lineage level was realized by comparison with profiles deposited in the SITVITWEB database and in SITVIT2 and compared with the classification obtained with previous version of the spoligotype database (Table S1).

Based on comparison with SITVIT2, 83 SITs were encountered in 195 isolates (89.4%), while 10 SIT (n = 15) had Unknown profiles and 23 isolates showed to have Orphan patterns. For classification of the latter two pattern types, we used Spotclust (Table S1). Ninety five isolates (43.6%) were classified as belonging to the LAM clade, 76 (34.9%) as belonging to the T clade, 40 (18.3%) as belonging to the Haarlem clade, five to the EAI5 clade (2.29%) and one each to the X clade (0.45%) and to Beijing. Overall, T1 (n = 52/23.8%), LAM9 (n = 32/14.7%) and H3 (n = 24/11%) were the most frequent subclades, while SIT53 (n = 26/50%), SIT42 (n = 18/56.25%) and SIT50 (n = 16/72,7%) were the most frequent SITs.

Table 1 illustrates the difference in frequency of SIT clusters containing three or more isolates (at least 3%) in this study, versus their worldwide distribution (macro-regions and countries), as defined in the SITVIT2 database. We observed a significantly higher frequency of genotypes T1 SIT53 (11.9% vs 5.5%; p = 0,027, X^2^ = 3,49) and a lower frequency of LAM9 SIT42 (8.2% vs 12.5%; p = 0,0001; X^2^ = 15.82). In addition, SIT 3907 (T1), SIT 2498 (H3) and SIT 3908 (LAM2) have so-far only been observed in Brazil. Presently, we also observed five SITs, 136 (LAM5), 189 (T1), 268 (H3), 2107 (unknown) and 2322 (T2), that had not been reported before in Brazil. When comparing our results with the previous and the present version of the SITVIT database, we noticed some differences in classification (Table S1). Thirty-six isolates could be classified using the new version and among these nine new SITs were observed: 3125 (LAM6), 3504 (LAM6), 3904 (LAM6) 3905 (T1) 3906 (T3), 3907 (T1), 3908 (LAM2; originally designated as SIT2560 Unknown in SITVITWEB), 3909 (LAM8) and 3910 (T1). In addition, nineteen orphan profiles were classified and SIT268, SIT1241 and SIT2539, without classification in SITVITWEB, were now classified respectively as H3, LAM9 and LAM1. In addition, some SIT showed changes in clade and/or subclade classification (SITs 73, 102, 159, 451, 742 and 777). Further information is detailed in Table S1.

**Table 1:** Description of clusters containing 3 or more isolates in this study and their worldwide distribution in the SITVIT2 database (interrogation made on September 25^th^ 2013).

SIT (Lineage) Octal Number; Spoligotype Description	Number (%) in study	% in study vs. database	Distribution in Regions with ≥3% of a given SITs *	Distribution in countries with ≥3% of a given SITs **
53 (T1) 777777777760771	26 (11.93)	0.41	EURO-W 15.65, AMER-N 13.48, AMER-S 12.45, EURO-S 9.41, EURO-N 7.48, ASIA-W 7.31, AFRI-S 4.97, AFRI-E 4.65, ASIA-E 4.23, AFRI-N 3.52, EURO-E 3.26, AMER-C 3.23	USA 13.2, FXX 7.88, BRA 5.54, ITA 5.33, ZAF 4.86, TUR 3.47, AUT 3.42, CHN 3.09
▪▪▪▪▪▪▪▪▪▪▪▪▪▪▪▪▪▪▪▪▪▪▪▪▪▪▪▪▪▪▪▪□□□□▪▪▪▪▪▪▪				
42 (LAM9) 777777607760771	18 (8.26)	0.54	AMER-S 29.7, AMER-N 11.85, EURO-S 11.31, EURO-W 9.46, AFRI-N 8.57, EURO-N 4.9, AMER-C 3.55, AFRI-E 3.55, AFRI-S 3.16	BRA 12.51, USA 11.85, COL 7.61, MAR 7.05, ITA 6.54, FXX 5.05, ESP 3.34, VEN 3.31, ZAF 3.16
▪▪▪▪▪▪▪▪▪▪▪▪▪▪▪▪▪▪▪▪□□□□▪▪▪▪▪▪▪▪□□□□▪▪▪▪▪▪▪				
50 (H3) 777777777720771	16 (7.34)	0.47	AMER-S 18.16, EURO-W 17.54, AMER-N 17.54, EURO-S 11.53, EURO-E 5.51, EURO-N 5.45, AFRI-N 4.25, AFRI-S 4.04, CARI 3.48, ASIA-W 3.21	USA 17.51, BRA 7.52, FXX 6.87, AUT 6.07, ITA 5.43, ESP 5.43, PER 4.72, ZAF 4.04, CZE 3.66
▪▪▪▪▪▪▪▪▪▪▪▪▪▪▪▪▪▪▪▪▪▪▪▪▪▪▪▪▪▪□▪□□□□▪▪▪▪▪▪▪				
SIT64 (LAM6) 777777607560771	6 (2.75)	1.6	AMER-S 49.33, AMER-N 25.07, EURO-W 6.4, EURO-S 4.8, ASIA-W 3.2	BRA 38.4, USA 25.07, GUF 6.13, PRT 3.73
▪▪▪▪▪▪▪▪▪▪▪▪▪▪▪▪▪▪▪▪□□□□▪▪▪▪□▪▪▪□□□□▪▪▪▪▪▪▪				
SIT47 (H1) 777777774020771	5 (2.29)	0.32	EURO-W 20.29, AMER-N 15.51, EURO-S 13.47, AMER-S 12.64, EURO-N 10.47, EURO-E 7.21, ASIA-W 4.02, AFRI-N 3.64	USA 15.19, ITA 8.23, AUT 8.04, BRA 7.34, FXX 6.64, FIN 6.0, CZE 3.77, ESP 3.57, SWE 3.38, PER 3.06
▪▪▪▪▪▪▪▪▪▪▪▪▪▪▪▪▪▪▪▪▪▪▪▪▪□□□□□□▪□□□□▪▪▪▪▪▪▪				
SIT60 (LAM4) 777777607760731	5 (2.29)	1.17	AFRI-S 38.08, AMER-S 21.5, EURO-W 7.24, AFRI-N 7.01, AFRI-W 6.31, EURO-S 5.61, AMER-N 4.4	ZAF 38.08, BRA 14.72, FXX 5.14, USA 4.44, MAR 4.44, GMB 3.97, ITA 3.74, VEN 3.51
▪▪▪▪▪▪▪▪▪▪▪▪▪▪▪▪▪▪▪▪□□□□▪▪▪▪▪▪▪▪□□□□▪▪▪□▪▪▪				
SIT73 (T) 777737777760731	5 (2.29)	1.94	EURO-S 17.05, EURO-W 13.95, AMER-N 13.95, AMER-S 11.24, AFRI-S 10.85, AFRI-E 7.36, ASIA-E 4.65, AMER-C 3.88	ITA 14.34, USA 13.95, ZAF 10.85, FXX 7.75, BRA 6.59, CHN 4.65, MOZ 3.88, MEX 3.1
▪▪▪▪▪▪▪▪▪▪▪▪□▪▪▪▪▪▪▪▪▪▪▪▪▪▪▪▪▪▪▪□□□□▪▪▪□▪▪▪				
4 (Unknown) 000000007760771	4 (1.83)	1.08	EURO-S 16.49, AMER-S 14.32, AMER-N 12.43, EURO-W 10.81, ASIA-W 10.54, AFRI-S 8.11, AFRI-E 8.11, EURO-N 6.22, EURO-E 5.68	USA 9.73, ITA 8.92, BRA 8.92, ZAF 8.11, TUR 7.84, BGR 5.41, ETH 4.32, FXX 4.05, ALB 4.05
□□□□□□□□□□□□□□□□□□□□□□□□▪▪▪▪▪▪▪▪□□□□▪▪▪▪▪▪▪				
SIT17 (LAM2) 677737607760771	4 (1.83)	0.6	AMER-S 59.58, AMER-N 17.95, CARI 8.9, EURO-S 5.58	BRA 28.36, VEN 27.0, USA 17.95, ESP 4.37, HTI 3.32, GLP 3.32
▪▪□▪▪▪▪▪▪▪▪▪□▪▪▪▪▪▪▪□□□□▪▪▪▪▪▪▪▪□□□□▪▪▪▪▪▪▪				
SIT33 (LAM3) 776177607760771	4 (1.83)	0.35	AFRI-S 28.76, AMER-S 25.31, AMER-N 14.07, EURO-S 12.57, EURO-W 7.26, AMER-C 4.78	ZAF 28.76, USA 14.07, BRA 12.04, ESP 7.88, PER 6.02, ARG 5.04, FXX 4.69, ITA 4.07, HND 3.81
▪▪▪▪▪▪▪▪□□□▪▪▪▪▪▪▪▪▪□□□□▪▪▪▪▪▪▪▪□□□□▪▪▪▪▪▪▪				
SIT3907 (T1) 777703777760631	4 (1.83)	100	AMER-S 100	BRA 100
▪▪▪▪▪▪▪▪▪▪▪▪□□□□▪▪▪▪▪▪▪▪▪▪▪▪▪▪▪▪□□□□▪▪□□▪▪▪				
SIT306 (T1) 777777601560771	3 (1.38)	11.54	EURO-W 38.46, AMER-N 26.92, AMER-S 19.23, EURO-N 7.69, AFRI-M 7.69	USA 26.92, BEL 26.92, BRA 15.39, FXX 11.54, GBR 7.69, CAF 7.69, GUF 3.85
▪▪▪▪▪▪▪▪▪▪▪▪▪▪▪▪▪▪▪▪□□□□□□▪▪□▪▪▪□□□□▪▪▪▪▪▪▪				
SIT828 (LAM4) 377777607760731	3 (1.38)	9.38	AMER-S 90.63, EURO-S 3.13, AFRI-W 3.13, AFRI-S 3.13	BRA 84.38, PRY 6.25, ZAF 3.13, ITA 3.13, GNB 3.13,
□▪▪▪▪▪▪▪▪▪▪▪▪▪▪▪▪▪▪▪□□□□▪▪▪▪▪▪▪▪□□□□▪▪▪□▪▪▪				
SIT1711 (LAM2) 677337607760771	3 (1.38)	37.5	AMER-S 75.0, EURO-S 12.5, AMER-C 12.5	BRA 37.5, VEN 25.0, MEX 12.5, ESP 12.5, COL 12.5,
▪▪□▪▪▪▪▪▪□▪▪□▪▪▪▪▪▪▪□□□□▪▪▪▪▪▪▪▪□□□□▪▪▪▪▪▪▪				
SIT2498 (H3) 777760077620771	3 (1.38)	27.27	AMER-S 100	BRA 100
▪▪▪▪▪▪▪▪▪▪▪▪▪▪□□□□□□□▪▪▪▪▪▪▪▪□□▪□□□□▪▪▪▪▪▪▪				
SIT3908 (LAM2) 677730207760771	3 (1.38)	75	AMER-S 100	BRA 100
▪▪□▪▪▪▪▪▪▪▪▪□▪▪□□□□▪□□□□▪▪▪▪▪▪▪▪□□□□▪▪▪▪▪▪▪				

*Worldwide distribution is reported for regions with more than 3% of a given SITs as compared to their total number in the SITVIT2 database. The definition of macro-geographical regions and sub-regions (http://unstats.un.org/unsd/methods/m49/m49regin.htm) is according to the United Nations; Regions: AFRI (Africa), AMER (Americas), ASIA (Asia), EURO (Europe), and OCE (Oceania), subdivided in: E (Eastern), M (Middle), C (Central), N (Northern), S (Southern), SE (South-Eastern), and W (Western). Furthermore, CARIB (Caribbean) belongs to Americas, while Oceania is subdivided in 4 sub-regions, AUST (Australasia), MEL (Melanesia), MIC (Micronesia), and POLY (Polynesia). Note that in our classification scheme, Russia has been attributed a new sub-region by itself (Northern Asia) instead of including it among rest of the Eastern Europe. It reflects its geographical localization as well as due to the similarity of specific TB genotypes circulating in Russia (a majority of Beijing genotypes) with those prevalent in Central, Eastern and South-Eastern Asia.

**The 3 letter country codes are according to http://en.wikipedia.org/wiki/ISO_3166-1_alpha-3; countrywide distribution is only shown for SITs with ≥3% of a given SITs as compared to their total number in the SITVIT2 database.

### Classification based on MIRU-VNTR analysis

The 218 isolates were also submitted to 24 loci MIRU-VNTR typing with the objective to confirm or not spoligotype-based classification of existing and new sublineages and to correct eventual miss classification that could have occurred using the latter technique. We observed 208 different patterns and seven clusters with two to four strains (clustering rate = 1.37%) (Figure S1). The allelic diversity of each MIRU-VNTR locus for 218 isolates was evaluated and classified as either highly (HGDI>0.6), moderately (0.6≤0.3) and poorly discriminative (HGDI<0.3), according to Sola *et al*. [15], as summarized in Table 2. Eight loci were highly discriminatory (QUB4156, QUB11, MTUB04, MIRU10, MIRU40, MIRU 23, MIRU26 and MIRU21) ten were moderate (MTUB30, MTUB39, MIRU16, ETR-C, ETR-A, QUB26, MTUB34, MIRU31, MIRU29 AND ETR-B) and six were poorly discriminative (MIRU24, MIRU29, MIRU04, MIRU27, MIRU20 and MIRU2). In the Table 2, we can observe a difference in discriminatory power of some loci according to lineage by Spoligotyping.

**Table 2:** Allelic diversity of 24 MIRU-VNTRs loci on 218 *Mycobacterium tuberculosis* strains isolated from pulmonary tuberculosis patients in Rio de Janeiro, Brazil.

	Genotype families by Spoligotyping			
MIRU-VNTR *loci*	LAM	H	T	All families
MIRU02	0.362	0.148	0.353	0.325
MIRU04	0.061	0.153	0.225	0.139
MIRU10	0.56	0.687	0.692	0.692
MIRU16	0.636	0.243	0.439	0.544
MIRU20	0.081	0.499	0.24	0.27
MIRU23	0.541	0.529	0.584	0.688
MIRU24	0.021	0.101	0.081	0.071
MIRU26	0.723	0.559	0.687	0.639
MIRU27	0.314	0.145	0.105	0.227
MIRU31	0.429	0.357	0.51	0.445
MIIRU39	0.138	0.251	0.027	0.124
MIRU40	0.517	0.623	0.76	0.689
ETR-A	0.14	0.417	0.625	0.537
ETR-B	0.407	0.237	0.396	0.389
ETR-C	0.357	0.447	0.573	0.541
MTUB04	0.612	0.53	0.68	0.716
MTUB21	0.436	0.587	0.673	0.624
MTUB29	0.253	0.432	0.266	0.397
MTUB30	0.269	0.494	0.652	0.591
MTUB34	0.617	0.305	0.617	0.452
MTUB39	0.391	0.573	0.391	0.577
QUB11	0.697	0.702	0.697	0.737
QUB26	0.384	0.63	0.384	0.522
QUB4156	0.842	0.842	0.842	0.84

**Figure 1:**
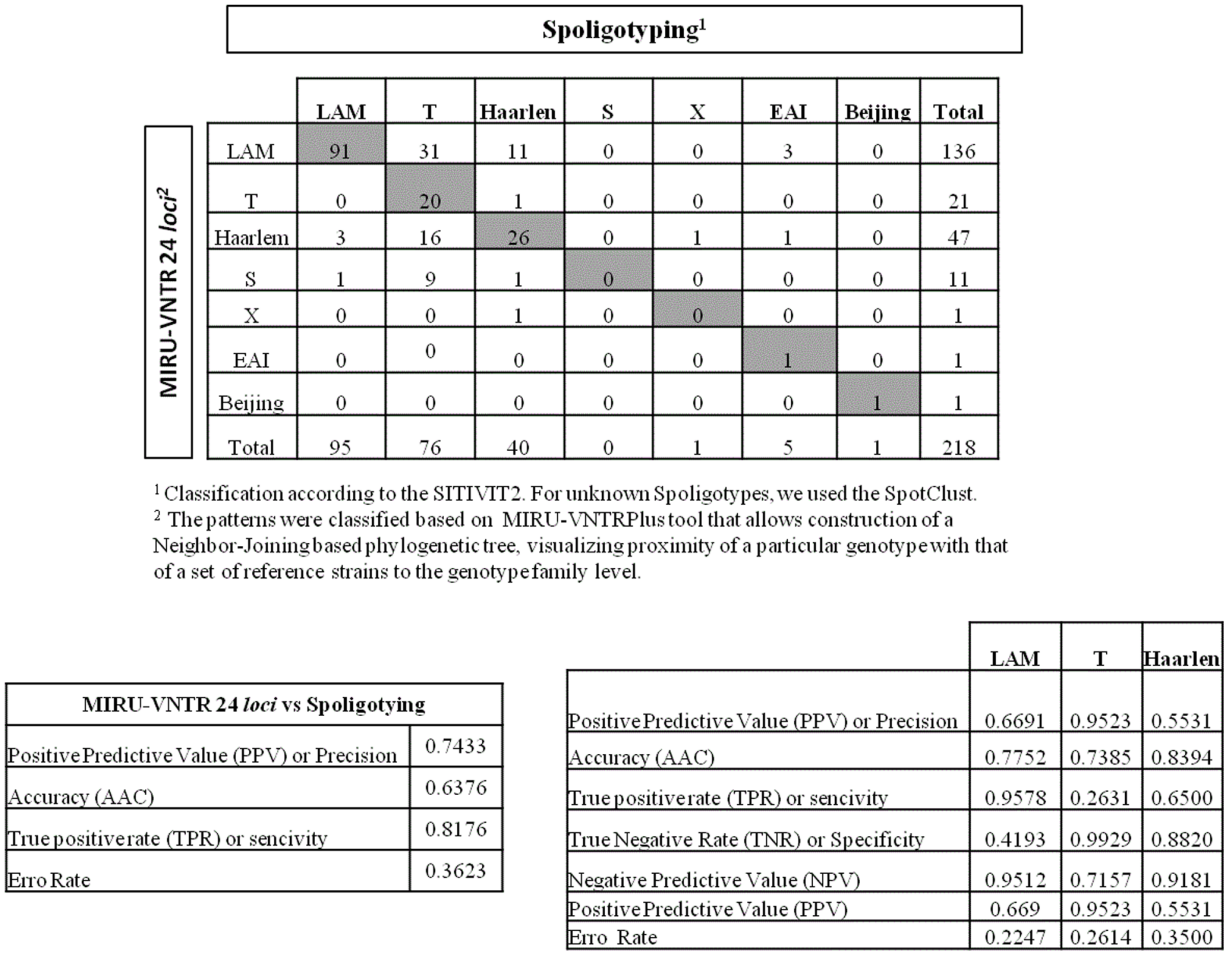
Confusion Matrix comparing the classifications obtained by Spoligotyping and MIRU-VNTR. ^1^ Classification according to SITIVIT2. For unknown Spoligotypes, we used SpotClust. ^2^ Patterns were classified based on a VNTR*plus* database that allows construction of a Neighbor-Joining based phylogenetic tree, visualizing proximity of a particular genotype with that of a set of reference strains to the genotype family level.

The patterns were classified based on the database tool that allows construction of a Neighbor-Joining based phylogenetic tree, visualizing proximity of a particular genotype with that of a set of reference strains to the genotype family level. One hundred thirty-six isolates (62.4%) were classified as LAM (127 patterns), 47 (21.5%) as Haarlem (45 patterns), 21 (9.6%) as T (21 patterns), 11 (5%) as S (11 patterns), and one isolate (0.45%) each as Beijing and EAI. Considerable differences were observed between Spoligotyping and 24 MIRU-VNTR loci-based classifications, even after excluding eventual small typing errors by repeating the assays. The differences observed between the two classifications are presented in the table 3 and in the Confusion Matrix (Figure 1). The precision, accuracy, sensitivity and error rate were respectively 0.74, 0.64, 0.82 and 0.36. The T lineage showed the highest incongruence rate related to classification (sensitivity = 0.26).

**Table 3:** Frequencies of strains according to classification by Spoligotyping (SITVIT2) and 24 loci MIRU-VNTR (MIRUVNTRPlus).

Lineage	Spoligotyping (%)		24 loci MIRU-VNTR (%)	
LAM	95	(43.58)	136	(62.39)
T	76	(34.86)	21	(9.63)
H	40	(18.35)	47	(21.56)
S	0	0	11	(5.05)
X	1	(0.46)	1	(0.46)
EAI	5	(2.29)	1	(0.46)
BEIJING	1	(0.46)	1	(0.46)

### Combined analysis of 24 MIRU-VNTR and spoligotyping

#### The LAM family

For better understanding of the population structure of the Mtb strains, dendrograms of spoligotyping and 24 MIRU-VNTRs, either separately or as combined patterns were constructed using BioNumerics. Among the 136 (62.4%) isolates that were classified as LAM based on MIRU-VNTR analysis, 99 (72.8%) exhibited the characteristic LAM spoligotype profile with absence of spacers 21 to 24 and 33–36. Among these however, four spoligotype isolates were designated to the T clade by SITVIT2 (SITs: 306/n = 3, 1688/n = 1, 3905/n = 1) and one as T2/orphan. Another thirty-seven strains (27.2%) defined as LAM by MIRU-VNTRs did not present the lack of spacers 21–24 and were therefore designated as “LAM-like”. Among isolates initially classified as LAM by spoligotyping (n = 95), 91 were confirmed as LAM (95.8%/TPR = 0.96) by 24 loci MIRU-VNTR (Figure 1). Six spoligotypes referred to as “Unknown” in SITVIT2 (SITs 132/n = 1; 1952/n = 1; 2107/n = 2; 2511/n = 2; 2535/n = 1 and 2548/n = 1) were also classified as LAM. Using SpotClust, SIT132 and SIT2511 were classified as EAI and SIT1952 as Haarlem. All Orphans patterns classified as LAM by SITVIT2 (n = 10) were also classified as LAM according to the MIRU-VNTR based phylogenetic tree.

Signatures based on MIRU copy number for “real” LAM (n = 99) types showed that the majority had two copies of MIRU04 (94; 95%) and two MIRU20 (n = 96; (97%), one copy of MIRU24 (n = 91; 92%), two copies of MIRU39 and ETR-A (n = 93; 94%) and 86 (87%) one copy of MTUB30. This indicates that the 221221 combination of these six alleles is representative for LAM strains, except for LAM3, presenting two copies of MTUB30 (221222).

#### The “LAM-like” isolates

The thirty-seven isolates that did not present the typical LAM spoligotype signature (absence of spacers 21 to 24 and 33–36) were positioned within the LAM branch in the neighbor-joining tree using MIRU-VNTR*Plus* and were conveniently designated as “LAM-like”. These had been classified by spoligotyping earlier as T (n = 24), H (n = 10) and three “Unknown” patterns (SIT2511/n = 2 and SIT1952/n = 1; classified by SpotClust as EAI and H1 respectively). Ninety-one percent of these strains showed one copy of MIRU24 and two copies of MIRU04. Intriguingly, when including both 24 MIRU-VNTRs and spoligotypes for tree building, these isolates are localized among those classified as T and H.

#### The T family

Among the isolates initially classified as T by spoligotyping, only 26.3% (20/76) was confirmed by MIRU-VNTR 24 loci (TPR: 0.2631) (Table 3 and figure 1). By a Neighbor-Joining based phylogenetic tree, 31 of these isolates (40.9%) were positioned in LAM branch, 16 (21%) in the Haarlem branch and 9 (11.8%) in the S branch. The MIRU-VNTR characteristic signature for the T lineage confirmed by both techniques, was one copy of MIRU24 (95.8%), two copies each of MIRU02 (95.8%), MIRU04 (87.5%), MIRU20 (95.8%), MIRU39 (100%) and three copies each of MIRU27 (87.5%) and MTUB34 (87.5%). Unlike LAM isolates that present a single copy of MTUB30, isolates of the T family presented two or four copies of this allele.

#### The Haarlem family

Forty-seven isolates were classified as belonging to the H family representing 21.5% of all isolate, 85% of the these isolates initially classified as Haarlem by Spoligotyping were confirmed by MIRU-VNTRs 24 *loci* (TPR = 0.65, TNR = 0,88) (Table 3 and figure 1). Among these isolated 16 (34%) presented spoliotype profile compatible with T family, 3 (6.4%) with LAM family and 2 (2% each) isolates, respectively compatible with X and EAI. The MIRU-VNTRs characteristic for Haarlem are two copies of MIRU02 (100%) and MIRU39 (89.1%) and three copies of MTUB34 (100%), MIRU16 (91.3%), MIRU27 (97.8%), ETR-A (95.6%) and ETR-C (91.3%). Different from LAM isolates having a single copy of MTUB30, H isolates present either three or four copies of this MIRU. Combined analysis of the MIRU-24 and spoligotyping showed that isolates of genotype H1 (absences of spacers 26–31) have two copies of MIRU04, ETR-B and MTUB29 and three copies of MIRU16, MIRU31, ETR-A and ETR-C. Isolates of genotype H3 (absences of spacer 31) share two copies of MIRU02, ETR-C, MIRU4 and MTUB29 and three copies of MIRU27, ETR-A, ETR-B and MTUB34.

#### The S family

Eleven isolates were classified as belonging to the S family and four of these exhibit SIT4, that in SpolDB4 was classified as LAM3-S Convergent, having absence of spacers 1–24 and 33–34 (The characteristic pattern of *S* family is absence of spacers 9–10, and 33–34). Among the others isolates, we observed SITs 53/n = 1, 102/n = 1, 378/n = 1, 2500/n = 1, 3907/n = 2 and 3909/n = 1; only two of these had the characteristic of S family (SIT3909/LAM8 and SIT2500/Unknown by SITVIT2 and H1 by Spotclust). Isolates of the S family shared their copy number in six loci, being one copy of MIRU24 and MTUB 21, two copies each of MIRU20 and MIRU39 and three copies MIRU10 and MIRU27.

### RD^Rio^, RD174 and *fbpC* ^103^ analysis

The genotypes defined by the presence of RD^Rio^, RD174 and SNP *fbp*C^103^ were added to the classification based on 24-MIRU-VNTR typing (Table S2). Thirty-six isolates (16.5%) were excluded from the final analysis either because of showing genotypes suggestive for multiple infection of because of failure in at least one of the three genotype procedures. The results of 182 strains are summarized in Table 3 and for simplification of interpretation, we defined three groups, being LAM (n = 77), LAM-like (n = 33) and non-LAM (n = 72) as determined by spoligotyping and MIRU-VNTR typing.

The SNP *fbpC* ^103^ was observed in 107 (58.8%) of the isolates, including 97.3% (74/77) of LAM, 69.6% (23/29) of “LAM-like” and 13.9% (10/72) of non-LAM. The total frequency of RD^Rio^ was 11% (20/182), with 20.7% (16/77) among LAM, 6.0% (2/29) among “LAM-like” and 2.7% (2/72) among non-LAM isolates. The overall frequency of RD174 was 15.3% (28/182), being 33.7% (25/77) in the LAM genotype and 6.8% (2/29) in “LAM-like”. All LAM/RD^Rio^ isolates presented the SNP *fbpC* ^103^ (Table 4 and Table 5), but not all were RD174; two isolates classified as LAM2 SIT3908 were not had RD174 (isolated from different patients). The frequency of RD174 (n = 25) was therefore higher than that of RD^Rio^ (32.5% vs 20.7%; p = 0.053 and X^2^ = 2,69) in LAM. Eleven LAM isolates (14.3%) presented RD174 but not RD^Rio^, all were positive for the *fbpC* ^103^.

**Table 4:** Descripition of RD^Rio^ by 24 loci MIRU-VNTRs, *fbpC* ^103^ and RD174.

									24 MIRU-VNTRs																							
									MIRU												ETR			MTUB						QUB		
									12 MIRU-VNTRs**												Additional 12 MIRU-VNTRs											
Strain	Year	Spoligotype Description	SITVIT2	SIT2	Lineage*	RD^RIO^	RD174	*fbpC^103^*	2	2	4	2	2	6	1	5	3	3	2	1	2	1	4	4	3	4	1	3	2	3	4	2
									2	4	10	16	20	23	24	26	27	31	39	40	A	B	C	4	21	29	30	34	39	11	26	4156
C0537A2	2009	▪▪▪▪▪▪▪▪▪▪▪▪▪▪▪▪▪▪▪▪□□□□▪▪▪▪▪▪▪▪□□□□▪▪▪▪▪▪▪	LAM9	42	**LAM**	+	+	+	1	**2**	**4**	3	**2**	**6**	**1**	4	**3**	3	**2**	**1**	*2*	*1*	*4*	3	*3*	*4*	*1*	5	2	*2*	7	3
C0996	2008	▪▪▪▪▪▪▪▪▪▪▪▪▪▪▪▪▪▪▪▪□□□□▪▪▪▪▪▪▪▪□□□□▪▪▪▪▪▪▪	LAM9	42	**LAM**	+	+	+	**2**	**2**	3	2	**2**	**6**	**1**	4	**3**	3	**2**	**1**	*2*	*1*	*4*	4	*3*	5	*1*	5	2	4	5	*2*
C2105	2008	▪▪▪▪▪▪▪▪▪▪▪▪▪▪▪▪▪▪▪▪□□□□▪▪▪▪▪▪▪▪□□□□▪▪▪▪▪▪▪	LAM9	42	**LAM**	+	+	+	**2**	**2**	2	2	**2**	**6**	**1**	4	**3**	3	**2**	**1**	*2*	*1*	*4*	4	*3*	*4*	*1*	6	2	4	*	*2*
C1231A2	2008	▪▪▪▪▪▪▪▪▪▪▪▪▪▪▪▪▪▪▪▪□□□□▪▪▪▪▪▪▪▪□□□□▪□□□□□□	LAM4	1106	**LAM**	+	+	+	**2**	**2**	**4**	1	**2**	**6**	**1**	3	2	3	**2**	**1**	*2*	*1*	*4*	4	*3*	*4*	*1*	*3*	2	*3*	1*6	*2*
C2314	2008	▪▪▪▪▪▪▪▪▪▪▪▪▪▪▪▪▪▪▪▪□□□□▪▪▪▪□▪▪▪□□□□▪▪▪▪▪▪▪	LAM6	64	**LAM**	+	+	+	**2**	**2**	3	1	**2**	**6**	**1**	4	2	3	**2**	**1**	*2*	*1*	*4*	0	*3*	*4*	*1*	5	0	*3*	8	*2*
C0097	2009	▪▪▪▪▪▪▪▪▪▪▪▪▪▪▪▪▪▪▪▪□□□□▪▪□□□□□□□□□□□□□□▪▪▪	Unknow	132	**LAM**	+	+	+	**2**	**2**	3	1	**2**	**6**	**1**	4	2	3	**2**	**1**	*2*	*1*	*4*	3	*3*	*4*	*1*	4	2	2	8	*2*
C6689	2008	▪▪▪▪▪▪▪▪▪▪▪▪▪▪▪▪▪▪▪□□□□□▪▪▪▪▪▪▪▪□□□□▪▪▪▪▪▪▪	LAM9	1800	**LAM**	+	+	+	**2**	**2**	**4**	3	**2**	**6**	**1**	**5**	3	2	1	3	*2*	2	*4*	*4*	*3*	6	*1*	*3*	2	2	7	*2*
C6582	2008	▪▪▪▪▪▪▪▪▪▪▪▪▪▪▪▪▪▪□▪□□□□▪▪▪▪▪□□□□□□□□▪▪□▪▪▪	LAM6	1066	**LAM**	+	+	+	**2**	**2**	2	1	**2**	4	**1**	4	2	**3**	**2**	**1**	*2*	*1*	*4*	*4*	*3*	*4*	*1*	5	2	4	7	*2*
C0817	2008	▪▪▪▪▪▪▪▪▪▪▪▪□▪▪□□□□▪□□□□▪▪▪▪▪▪▪▪□□□□▪▪▪▪▪▪▪	LAM5	Orphan	**LAM**	+	+	+	**2**	**2**	4	**2**	**2**	5	**1**	**5**	**3**	**3**	**2**	**1**	*2*	2	*4*	*4*	*3*	*4*	*1*	2	2	*3*	4	*2*
C1893	2009	▪▪▪▪▪▪▪▪▪▪▪▪□□□□□□▪▪□□□□▪▪▪▪▪▪▪▪□□□□▪▪▪▪▪▪▪	LAM5	1337	**LAM**	+	+	+	**2**	**2**	2	**2**	**2**	**6**	**1**	**5**	**3**	**3**	**2**	**1**	*2*	*1*	*4*	*4*	*3*	*4*	*1*	4	1	2	1	*2*
C2559	2009	▪▪□▪▪▪▪▪▪▪▪▪□▪▪▪▪▪▪▪□□□□▪▪▪▪▪▪▪▪□□□□▪▪▪▪▪▪▪	LAM2	17	**LAM**	+	+	+	**2**	**2**	**4**	**2**	**2**	5	**1**	6	**3**	**3**	**2**	**1**	*2*	2	5	*4*	*3*	*4*	*1*	*3*	*2*	2	6	*2*
C3554	2009	▪▪□▪▪▪▪▪▪▪▪▪□▪▪▪▪▪▪▪□□□□▪▪▪▪▪▪▪▪□□□□▪▪▪▪▪▪▪	LAM2	17	**LAM**	+	+	+	**2**	**2**	**4**	**2**	**2**	5	**1**	6	**3**	**3**	**2**	**1**	*2*	2	*4*	*4*	*3*	*4*	*1*	*3*	*2*	2	6	*2*
C2020	2009	▪▪□▪▪▪▪▪▪▪▪▪□▪▪□□□□▪□□□□▪▪▪▪▪▪▪▪□□□□▪▪▪▪▪▪▪	LAM2	3908	**LAM**	+	−	+	**2**	**2**	**4**	**2**	**2**	5	**1**	**5**	**3**	**3**	**2**	**1**	*2*		*4*	*4*	*3*		*1*		*2*	*3*	*4*	*2*
C2046	2009	▪▪□▪▪▪▪▪▪▪▪▪□▪▪□□□□▪□□□□▪▪▪▪▪▪▪▪□□□□▪▪▪▪▪▪▪	LAM2	3908	**LAM**	+	−	+	**2**	**2**	**4**	**2**	**2**	5	**1**	**5**	**3**	**3**	**2**	**1**	*2*	2	*4*	*4*	*3*	*4*	*1*	2	*2*	*3*	*4*	*2*
C2017	2008	▪▪□▪▪▪▪▪▪▪▪▪□□▪□□□□▪□□□□▪▪▪▪▪▪▪▪□□□□▪▪▪▪▪▪▪	LAM2	Orphan	**LAM**	+	+	+	**2**	**2**	**4**	**2**	**2**	5	**1**	**5**	**3**	**3**	**2**	**1**	*2*	2	*4*	*4*	*3*	*4*	*1*	2	*2*	*3*	*4*	*2*
C2009	2009	▪▪□▪▪▪□▪▪▪▪▪▪▪▪▪▪▪▪▪□□□□▪▪▪▪▪▪▪▪□□□□▪▪▪▪▪▪▪	LAM1	2539	**LAM**	+	+	+	**2**	**2**	**4**	**2**	**2**	5	**1**	**5**	**3**	**3**	**2**	**1**	*2*	2	*4*	1	*3*	5	*1*	*3*	*2*	2	3	*2*
C1966	2008	▪▪▪▪▪▪▪▪▪▪▪▪▪▪▪▪▪▪▪▪▪▪▪▪▪▪▪▪▪▪□▪□□□□▪▪▪▪▪▪▪	H3	50	LAM-like	+	+	+	**2**	**2**	**4**	**2**	**2**	**6**	**1**	**5**	**3**	**3**	**2**	**1**	*2*	*1*	*4*	*4*	*3*	*4*	*1*	5	*2*	*3*	6	2
C0669	2009	▪▪▪▪▪▪▪▪▪□▪▪▪□▪▪▪▪▪▪□▪▪▪▪▪▪▪▪▪▪▪□□□□▪▪□□▪▪▪	T1	Orphan	LAM-like	+	−	−	**2**	**2**	**4**	*	**2**	5	**1**	5	**3**	**3**	**2**	**1**	3	*1*	*4*	1	4	*4*	*1*	*	2	2	8	*2*
C2142A2	2009	▪▪▪▪▪▪▪▪▪▪▪▪▪▪▪▪▪▪▪▪▪▪▪▪▪▪▪▪▪▪□▪□□□□▪▪▪▪▪▪▪	H3	50	H	+	−	−	3	**2**	**4**	3	1	2	**1**	2	**3**	**3**	**2**	2	3	2	3	2	2	2	4	3	3	4	*	2
C5817	2008	□□□□□□□□□□□□□□□□□□□□□□□□▪▪▪▪▪▪▪▪□□□□▪▪▪▪▪▪▪	Unknow	4	S	+	−	+	3	3	**4**	2	2	5	**1**	6	**3**	2	**2**	2	4	2	*4*	3	1	*4*	2	3	0	3	7	2

*Classification based on construction of phylogenetic tree using the N-J Algorithm and evaluating proximity of particular isolate with a set of 182 reference strains in the MIRU-VNTRPlus. Note: Strains with RD^Rio^ (LAM, Like-LAM and Non LAM).

**In bold the same number of copy of hypothetic Ancestral RD^Rio^ as suggested by lazzarini *et al*, 2007 [2] and n the probable number of copy of hypothetic Ancestral RD^Rio^ in the additional 12 *loci*.

**Table 5:** The frequency of *fbpC* ^103^, RD^Rio^ and RD174 in LAM, LAM-like and Non LAM isolates, as designated by spoligotyping and 24 MIRU-VNTR typing.

	LAM (%)	LAM-Like (%)	Non LAM (%)	Total Frequency (%)
SNP and RD	(n = 77)	(n = 29)	(n = 75)	(n = 182)
*fbpC* ^103^	74 (97.3)	23 (79.3)	10 (13.3%)	107 (58.8)
RD^Rio^	16 (20.7)	2 (6.9%)	2 (2.6%)	20 (11)
RD174	25 (32.5)	2 (6.9%)	1(1.3%)	28 (15.4)

**Figure 2:**
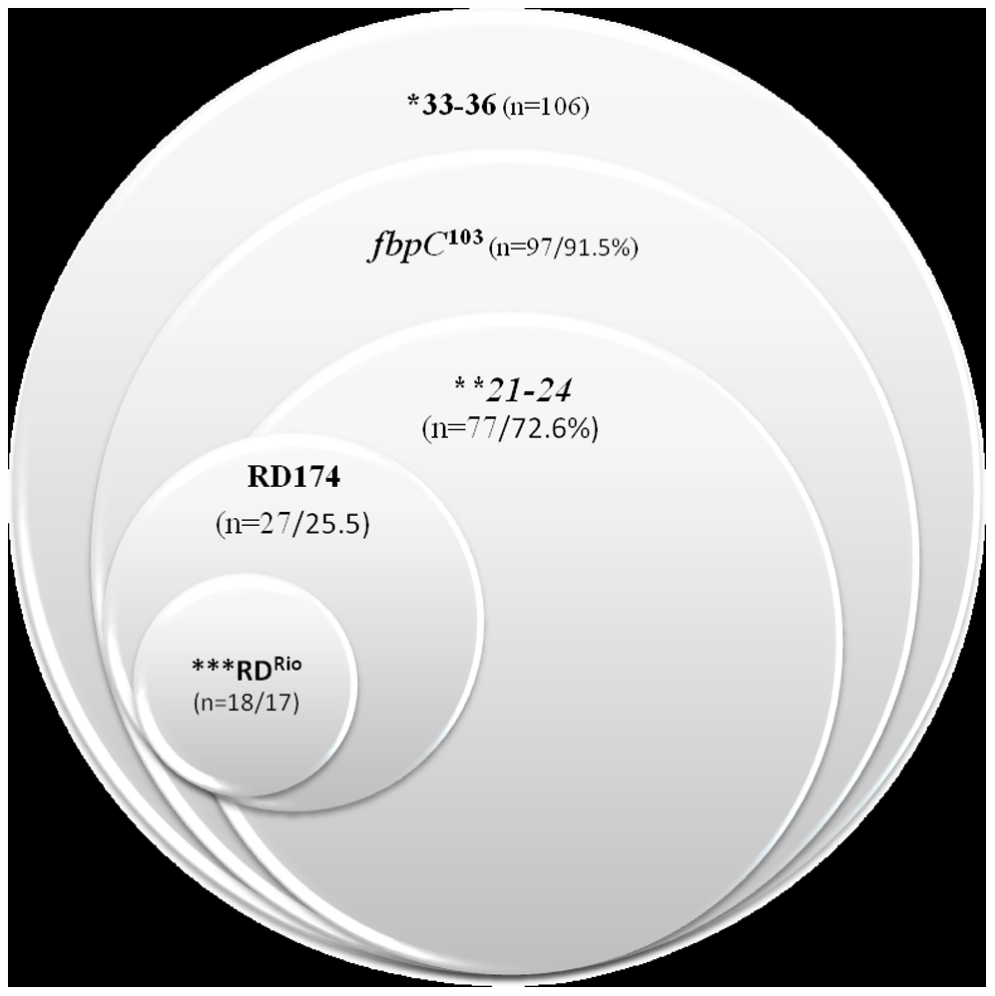
Venn diagram illustrating the different markers in isolated classified as LAM by 24 loci MIRU-VNTR. Notes: The Venn diagram was constructed based on LAM isolates defined by 24 loci MIURU-VNTR. The sizes of the circles is not proportional to the real frequency of these markers. Twenty isolates classified with LAM by MIRU-VNTRs were removed from the final analysis because of showing a mixed genotype or failure in at least one of the three genotype procedures. * absence of spacers 33–36 in the spoligotyping profile, ** absence of spacers 21–24 in the spoligotyping profile, ***Two isolates RD^Rio^ but not RD174 (LAM2 SIT3908).

The allelic diversity of the 24 loci MIRU-VNTR loci in LAM/RDRio isolates (Figure S2) showed that the copy number of combining of MIRU04, MIRU20, MIRU24, MIRU31, ETR-A, MTUB21 and MTUB30 loci was a signature of this genotype (2213231) (Table 4). In general, these loci present low variability in LAM, except for MTUB21, is being moderately variable in such isolates (table 2). Upon comparing 24 MIRU-VNTR signatures of LAM, LAM-like and non-LAM strains (Table 4), we observed two isolates that presented the hypothetical ancestral MIRU-VNTR signature (224226153321) for RD^Rio^ that was suggested by Lazzarini et al. (2007) [2]. One isolate (C2009) was classified as LAM1 SIT2539 and the other (C1966) as H3 SIT50 by spoligotyping and “LAM-like” by 24 MIRU-VNTR; both presented SNP *fbp*C^103^ and were deleted for RD^Rio^ and RD174. Among the LAM/RD^Rio^ strains, frequency of LAM subtypes as defined by spoligotyping using SITVIT2 was 31.5% of LAM2, 25% of LAM9, 12.5% each of LAM6 and LAM5 and 6.3% each of LAM4 and LAM1. Figure 2 is a graphical representation of these three markers in the LAM strains as defined by 24 loci MIRU-VNTRs.

## Discussion

Rio de Janeiro is the capital of the state of Rio de Janeiro, located in the southeast of Brazil, has a population of 16 million habitants, six and a half million of these living in the capital, being the second largest city and a major touristic attraction in Brazil (Census 2010 http://cidades.ibge.gov.br/xtras/perfil.php?lang=&codmun=330455 accessed 12/18/2013). According to the Ministry of Health, 11.639 new TB cases were recorded in 2011 in the state with an incidence of 72.3/100.000 habitants and the highest mortality rate (5.1/100.000 habitants) at the national level [1]. Rio de Janeiro city has strong economic and social contrasts, with a large portion of the population living in numerous suburbs, consisting of “communities”, urban areas where housing conditions, health, education and security are extremely precarious. These factors are directly related to the number of TB cases detected.

In the present study, we performed spoligotyping and 24 MIRU-VNTR typing and characterized a SNP in *fbpC* ^103^ and the status of RD174 and RD^Rio^, to decipher the population structure and the phylogenetic relationships of the MTBC strains of TB cases in this particular population that attended a single reference center in the city of Rio de Janeiro. *Mycobacterium tuberculosis* is classified into six phylogenetic lineages, each of which can be divided into sublineages [32]. Members of the Euro- American lineages represented by the LAM, H, T, S and X Spoligotyping families, which are genetically closely related are the most common in South America [19], [20], [21] and this was confirmed recently also in Brazil [22] and in different states of the country [8], [43], [44], [45], [46], [47] [48], but differences in the frequency of these families are observed, theses difference could also be due to differences in population and immigration history in each region or in period associated genotype frequencies or in differences in sample size. We here confirm the predominance of the Euro-American lineage (also known as lineage 4) with only two isolates classified as Indo-Oceanic lineage (EAI5) and East Asian lineage (Beijing), the first being more prevalent in east Africa, southeast Asia and in south India and the second in east Asia, Russia and South Africa. [32]. Recently, the frequency of such strains in Brazil was described as being less than 1% except for the higher frequency in the state of Pará, north Brazil [22].

The most prevalent families as defined by spoligotyping were LAM (43.6%), T (34.9%) and H (18.3%); however, classification based on 24-MIRU-VNTR and Neighbor-Joining based phylogenetic tree building using the database tool (www.miruvntrplus.org) showed considerable difference with that obtained by spoligotyping, being respectively 62.4%, 9.6% and 21.6% (error rate of 0.36). Among the isolates with discordant results, mostly isolates initially classified as T (n = 31), H (n = 11) and EAI (n = 3) by spoligotyping were classified as LAM by 24 MIRU-VNTRs typing, for isolates that did not show LAM prototype (absence of spacers 21–24 and 33–36), conveniently named LAM-like. Subsequently, 78.3% these isolates confirmed to be LAM by the presence of the LAM-specific SNP *fbpC* ^103^ [7], [38] and when considering the presence of this SNP as an absolute marker for LAM family the frequency of this lineage is 58.9%. We also have preliminary data showing that the supposed LAM-specific marker *ligB* ^404^ was observed in isolates that had been defined by spoligotyping as being T or H (data not shown). Very recently, Mokrousov et al. reported difference in classification of LAM strains as defined by spoligotyping and SNP analysis (*fbpC* ^103^ and *ligB* ^404^) [49] although both SNPs were previously validated in different collection of clinical isolates and reference strains as to be specific for the LAM family [7], [38].

It is now common knowledge that spoligotyping has limitations as a tool prediction of the exact phylogenetic relationships between strains of the MTBC, particularly in modern strains (Euro American, East Asian and Indian e East African) [16], [18], [24], [48], [49], [50], [51] [52], mainly due to homoplasy [16], [24]. The accuracy of the phylogenetic grouping by MIRU-VNTR is more exact than that of spoligotyping but depends on the number of loci included in the analysis [49] and classification errors are reduced when analyzing 24 loci [16]. Indeed, several authors have suggested that SNPs are more suitable than spoligotyping and MIRU-VNTR settings for phylogenetic classification [16], [38], [53], [54], [55], [56]. The *fbpC* ^103^ is described as a good marker for the LAM family [7], [38], [49], [57], [58], [59], and our original intention was to evaluate if strains RD^Rio^ was still prevalent in Rio de Janeiro as seen previously by Lazzarini in clinical isolates collected between 2002 and 2003 [2], for this purpose we believed only a marker that could differentiate LAM and non-LAM associated with the 24 MIRU-VNTR and Spoligotyping could be sufficient, however the scenario was observed more complex.

We here present the first data comparing classification by spoligotyping and MIRU-VNTRs and *fbpC* ^103^ in Euro American lineage prevalent in Rio de Janeiro and Brazil. Among these different types of markers, we observed four major groups: (i) strains classified as LAM by spoligotyping and MIRU-VNTRs 24 loci without the LAM-characteristic SNP *fbpC* ^103^, (ii) strains not classified as LAM by spoligotyping and MIRU-VNTRs 24 loci but carrying the *fbpC* ^103^ SNP, (iii) strains classified by spoligotyping as non-LAM but as LAM by MIRU-VNTRs 24 loci (LAM-like) and with SNP *fbpC* ^103^ and (iv), strains classified by spoligotyping as non-LAM and LAM by MIRU-VNTRs 24 loci (LAM-like) but not presenting the SNP *fbpC* ^103^. These different scenarios could be explained by convergent evolution of spoligotypes and of MIRU-VNTRs loci (even including 24 alleles) because a limited number of loci were evaluated that might evolve rapidly and therefore susceptible to pronounced convergence [53] and/or because the existence of ancestral progenitor of Euro- American lineages containing SNP *fbpC* ^103^. In addition, a possible important limitation of the current classification based on MIRU-VNTRs and their similarity with genotypes present in MIRU-VNTRplus is that, despite including well characterized strains, the database contains a limited number of strains that does not reflect the real genetic diversity of isolates belonging to the MTBC; another limitation of this study is that no additional specific SNPs for H, T, S and X and T were investigated. The Whole Genome Sequencing (WGS) is superior to conventional genotyping for MTBC [60] and has been used in areas not yet studied, from global (phylogeography) for site (transmission chains and diversity of circulating strains), for single patient (clonal diversity) and the bacterium itself (evolutionary studies) [61]. We intend to compare through WGS (developing) these isolates to a better comprehension of the evolution of lineages Euro American, such as the development of new methodologies that allow a more rapid and accurate typing.

Analyzing the data obtained in this study, the spoligotypes of the T family showed the largest number of divergent results when compared to classification by 24-MIRU (TPR = 0,26/TNR = 0,99). The prototype of the T family is characterized by the absence of spacers 33–36 only [48] and have been observed in almost every country, representing 20% of all isolates deposited in the database SITVITWEB. Despite the high frequency, this genotype family is still considered as “ill-defined” and includes non-monophyletic groups [54] [55]. In South America, the frequency of this family is 26.7% and in Brazil 18.6% (370/1991), with the T1 SIT53 subfamily being the most prevalent SIT [22], as observed also in this study. Here, among the 76 isolates classified as T by spoligotyping, only 21 (27.6%) were confirmed by 24 MIRU-VNTRs typing, the rest was reclassified as LAM (n = 31), S (n = 9) and H (n = 16). Interestingly, SIT53 was associated with mixed infection in a study conducted in South Africa, a country characterized by a high prevalence of TB [57] and Lazzarini *et al.* [62], using a computational approach, showed that this is the most frequent false spoligotype derived from mixed infections. However, among isolates with this SIT, we did not observe double signals during 24 MIRU typing indicative for mixed infections. We also observed that spoligotypes, indicative for the T family, were sometimes grouped with spoligotypes of the H family by MIRU-VNTR typing. This could be related with the fact that the prototype spoligotype defining T1 SIT53 and H3 SIT 50 differ only in the absence of the spacer 31. We also observed that in our population, the absence of spacer 31 is not crucial for classification as being either H or T; what differentiates between the two is the number of copies of ETR-A, ETR-B, ETR-C. The H3 subfamily is characterized by the combination 323 of these alleles while T strains present considerable diversity of these loci (one to three copies of ETR-A, two or three copies of ETR-B and three to five copies of ETR-C).

The RD nominated RD^Rio^ was first reported as new *M. tuberculosis* lineage in Rio de Janeiro in 2007, Lazzarini *et al.* [2] and is a deletion of 26.3 kb restricted to the LAM family and in particular, in subfamilies LAM1, LAM2, LAM4, LAM5, LAM6 and LAM9. This deletion affects 10 genes, including two genes encoding Proline-proline-glutamic Acid Proteins (PPE) [2] and association between RD^Rio^ strains and high prevalence may be related to virulence and/or adaptation specifies the Latin American and European population-based epidemiological and clinical characteristics; however, studies have proven to be inconclusive or contradictory [4], [63], [64]. The lineage RD^Rio^, was identified in different countries [5], [6], [7], [49] and in Brazil, has been described in different states, including Rio de Janeiro, Rio Grande do Sul, Minas Gerais, Espírito Santo and Rio Grande do Sul [3], [8], [63], [64]. The frequency ranges from 30 to 38% of isolates tested and was associated with MRD-TB [4], [63], [64] and with genotype clustering [5],[63], indicating a higher rate of recent infection and transmissibility. Another deletion, RD174, initially described as a marker for the LAM family and as a co-marker for RD^Rio^ [7], [8] was associated with high transmissibility [10].

The frequency of strains RD^Rio^ in the present study, with an overall frequency of 11% and 20.7% in LAM, is lower than that observed in other studies and even when including the eight isolates that showed mixed RD^Rio^/WT signals, resulting frequencies of 14.7% and 28.2% still lower than previously observed. This difference could be related with the relative low frequency of LAM1, LAM2, LAM5, LAM6 and LAM9 related subfamilies in our study sample. Earlier studies on classification of RD^Rio^ strains was based on genotypes defined by spoligotyping and/or 12 MIRUs only [2], [5], [6], [7], [8], [63], [64] and again, this is the first study that used 24 MIRU-VNTR typing to conduct a more detailed phylogenetic analysis. We verified the signature of MIRU-VNTR loci for RD^Rio^ and RD174, as compared with that of the hypothetical ancestral of RD^Rio^ as defined by 12 MIRUs [2] and that, besides sharing two copies of MIRU04 and MIRU20, they also share one copy of MIRU24, three copies of MIRU31, two copies of ETR-A, three copies of MTUB21 and one copy of MTUB30, yielding 2213231 as fingerprint for RD^Rio^. All LAM/RD174 isolates, with or without RD^Rio^, carried two copies of MIRU20 and ETRA and one copy of MTUB31. We observed that LAM3 has two copies of MTUB30 (2213232) and we propose that this subfamily that do not carry RD^Rio^, has evolved independently. We also observed RD^Rio^ in two isolates (2.6%) with a spoligotype not indicative for being LAM; a small number of such cases have also been observed by other research groups [5], [7], [64]. One of these isolates that had been classified by spoligotyping as H3 SIT53 was reclassified by 24 MIRU as being “LAM-Like” and called attention because it had the MIRU signature of the hypothetical ancestor of RD^Rio^ and carried the SNP *fbpC* ^103^ and RD174. Overall, we observed four scenarios: (i) isolates with the RD^Rio^ and RD174, (ii) isolates showing only RD^Rio^ (iii) isolates showing only RD174 and (iv) isolates that did not carry any of the deletions, suggesting that both markers evolved on different time points. This is different from earlier data [7] that claim that RD174 is an absolute co-marker for RD^Rio^ and therefore, studies that use the presence of RD174 to infer the presence of RD^Rio^ [8] may overestimate the frequency of the latter. In 2007, Lazzarini et al. [2] suggested that RD^Rio^ arose by homologous recombination between genes and although all neighboring sequences were identical, such event could have happened more than once. This suggests that the RD174 deletion occurred before RD^Rio^ but this needs to be confirmed as we also observed RD^Rio^ strains that had intact RD174. In Figure S3, we propose a possible evolution of members of the Euro-American lineages but with the limitation that the spoligotype defined lineages S, X and other are not represented and this concerns a sampling only from Rio de Janeiro.

In a recent study, Hill *et al.* (2012) [53], mentions the difficulty in studying the evolution of the Euro-American lineage (LAM, Haarlem, T, X and S) using spoligotyping due to the large number of IS*6110* copies in such strains that may result in IS*6110* mediated deletions in the DR locus. This might be the reason why bacterial evolution exclusively based on spoligotyping is not robust and the wide range of profiles reported as unclassified in SITVITWEB. Our approach, combining spoligotyping, MIRU-VNTRs, SNPs and RDs allowed the reclassification of 13 SITs that did not rank in SITVITWEB, allowing definition of 29 new spoligotypes and refine classification of isolates belonging to Euro-American lineage. Our data are also support the idea that absence of spacers 21–24 is not sufficient for classification as LAM and of spacer 31 to differentiate T and H, the latter indicative for subfamilies H3 SIT50 and T1 SIT53. Possible explanations are that ancestral lineages are currently circulating (plesiomorphic state) or that the isolates are suffering homoplasic evolution and reversion into the plesiomorphic state.

## Supporting Information

Figure S1 **Dendrogram constructed with BioNumerics software version 6.6 with the results of MIRU-VNTRs 24** ***loci*** **and spoligotyping by similarity coefficient for categorical data and the neighbor-joining algorithm.** 1st column (after spoligotypes): number of isolated label; 2nd column: patients who have more than one isolate in the study (n = 27) received numbering 1–27, and the different strains present the same numbering; 3rd column: International Spoliotype Types (SIT); 4th column: classification obtained through SITVITWEB (family and subfamily).
(DOC)

Figure S2 **Allelic diversity of the 24 loci MIRU-VNTR loci in LAM/RDRio isolates.**
(DOC)

Figure S3 **Possible evolution of** ***M. tuberculosis*** **lineage Euro-American (LAM, T and H) according to the markers analyzed in this study.** The Euro-American Ancestral evolved into two distinct groups: LAM ancestral and T/H ancestral, characterized by absences of spacers 33–36 and one copy of MIRU24 that is common to all Euro-American lineages. The ancestral LAM strains have *fbpC^103^* and this was the basis of LAM A (LAM9), with additional absence of spacers 21–24, one copy of MTUB30 and two copies of MIRU04 and ETR-A. LAM A on its turn is the basis for two other groups: LAM B (LAM9-LAM4, LAM1-LAM2-LAM5 and LAM6) and LAM C (LAM3). The LAM B evolved from LAM B1, characterized by a deleted RD174 and on its turn to LAM B2, with both deleted RD174 and RD^Rio^. The H/T Ancestral lineage is the origin of both groups H and T (difference only in MIRU-VNTR copies), showing absence of spacers 33–36; additional loss of spacer 31 led to subtype H A, observed in high frequency in this study.
(DOC)

Table S1 **Detailed genotyping results and associated demographic, epidemiologic and nomenclature information on 218 Mycobacterium tuberculosis strains isolated from pulmonary tuberculosis patients in Rio de Janeiro, Brazil.**
(XLS)

Table S2 **Detailed genotyping results of** ***fbpC*** **^103^, RD174 and RD^Rio^ on 182 M. tuberculosis strains isolated from pulmonary tuberculosis patients in Rio de Janeiro, Brazil.**
(XLS)
