# Supplementary figures and images for: Strain Classification of Mycobacterium tuberculosis Isolates in Brazil Based on Genotypes Obtained by Spoligotyping, Mycobacterial Interspersed Repetitive Unit Typing and the Presence of Large Sequence and Single Nucleotide Polymorphism
Source: PLoS One. 2014 Oct 14;9(10):e107747. doi: 10.1371/journal.pone.0107747 (PMC4196770; doi:10.1371/journal.pone.0107747)

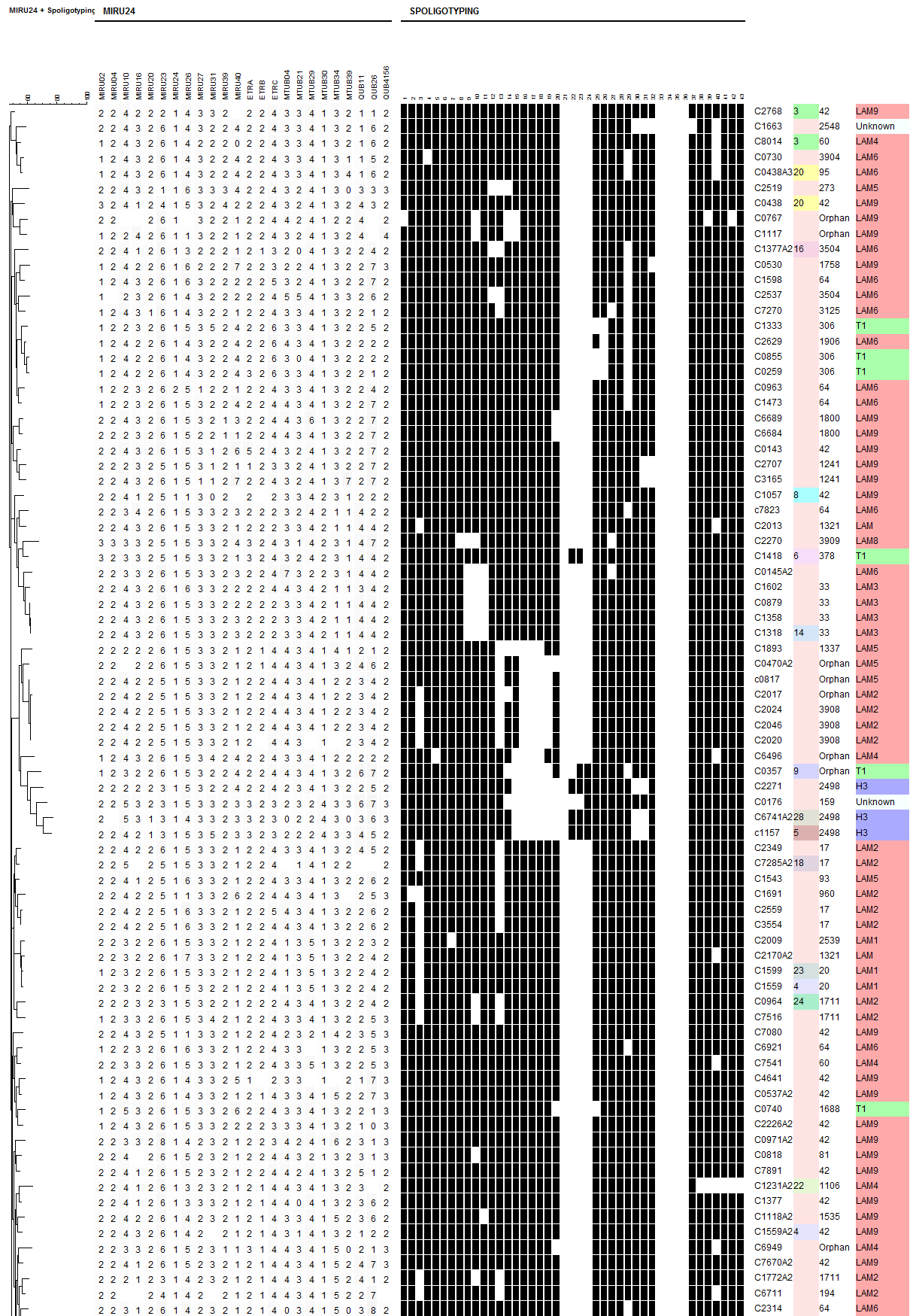
Fi

Figure S1.


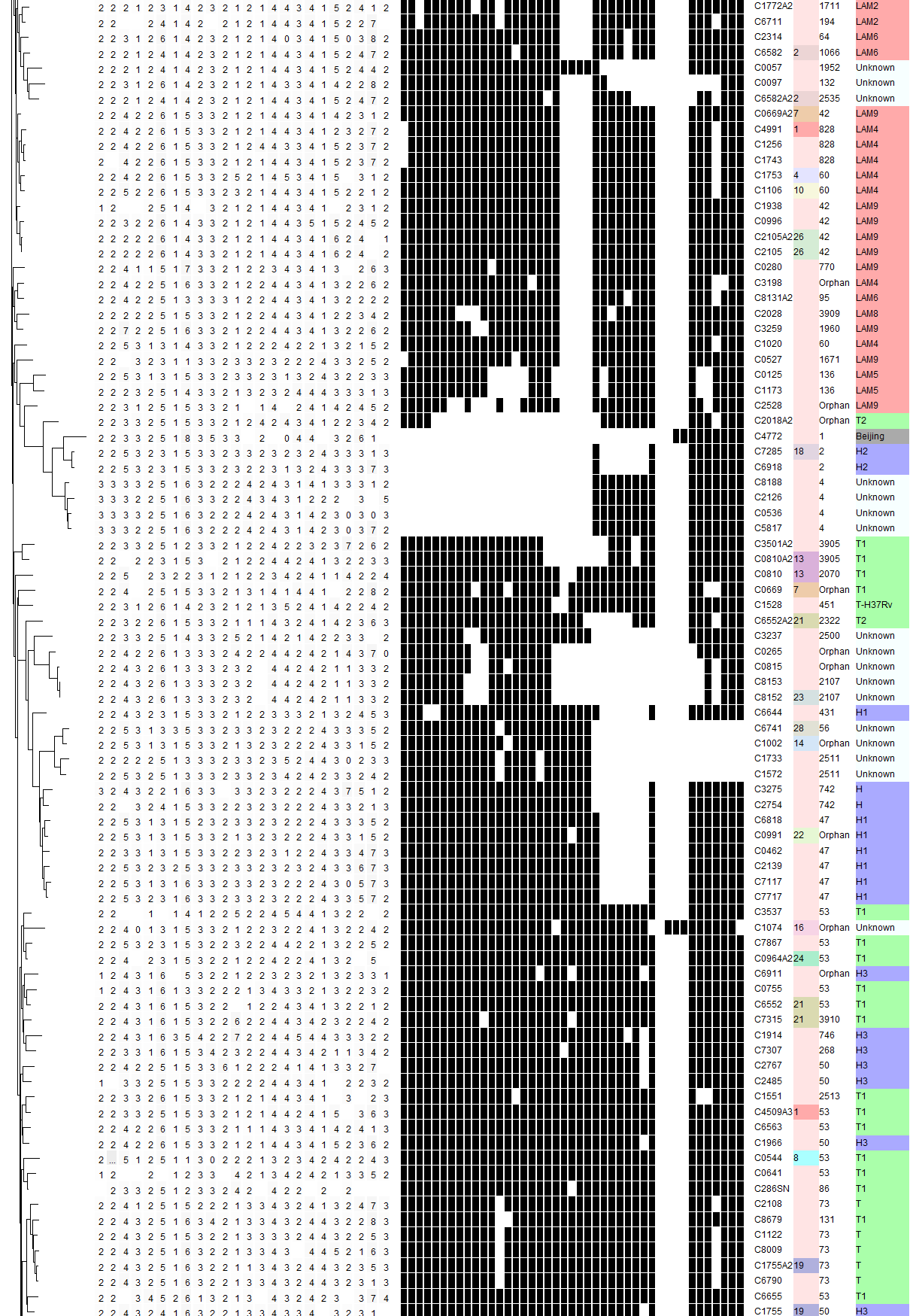


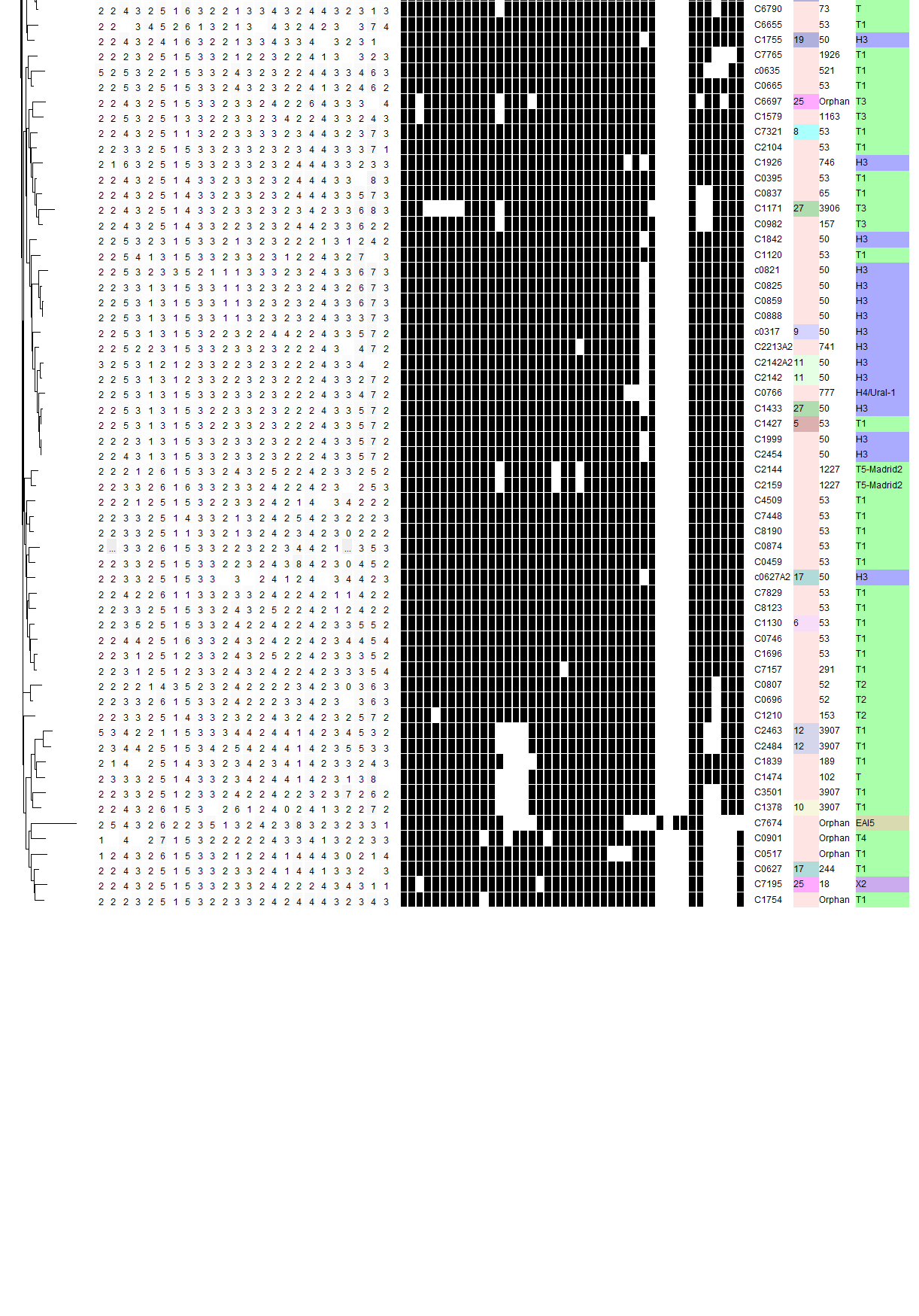

Supplement: Figure S1 — Dendrogram constructed with BioNumerics software version 6.6 with the results of MIRU-VNTRs 24 loci and spoligotyping by similarity coefficient for categorical data and the neighbor-joining algorithm. 1st column (after spoligotypes): number of isolated label; 2nd column: patients who have more than one isolate in the study (n = 27) received numbering 1–27, and the different strains present the same numbering; 3rd column: International Spoliotype Types (SIT); 4th column: classification obtained through SITVITWEB (family and subfamily). (DOC) [file pone.0107747.s001.doc]

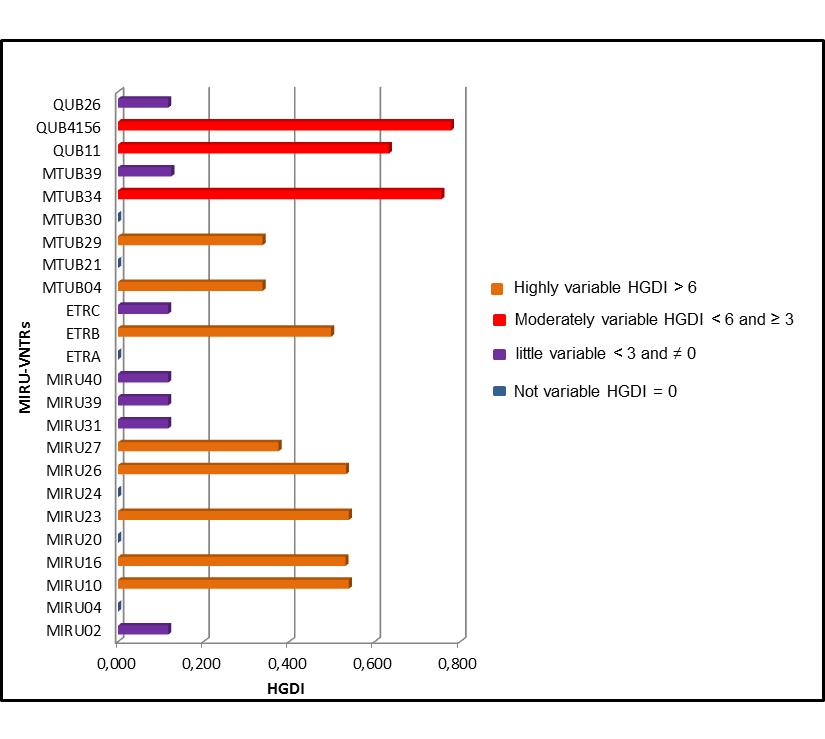
 Figure S2.

Supplement: Figure S2 — Allelic diversity of the 24 loci MIRU-VNTR loci in LAM/RDRio isolates. (DOC) [file pone.0107747.s002.doc]

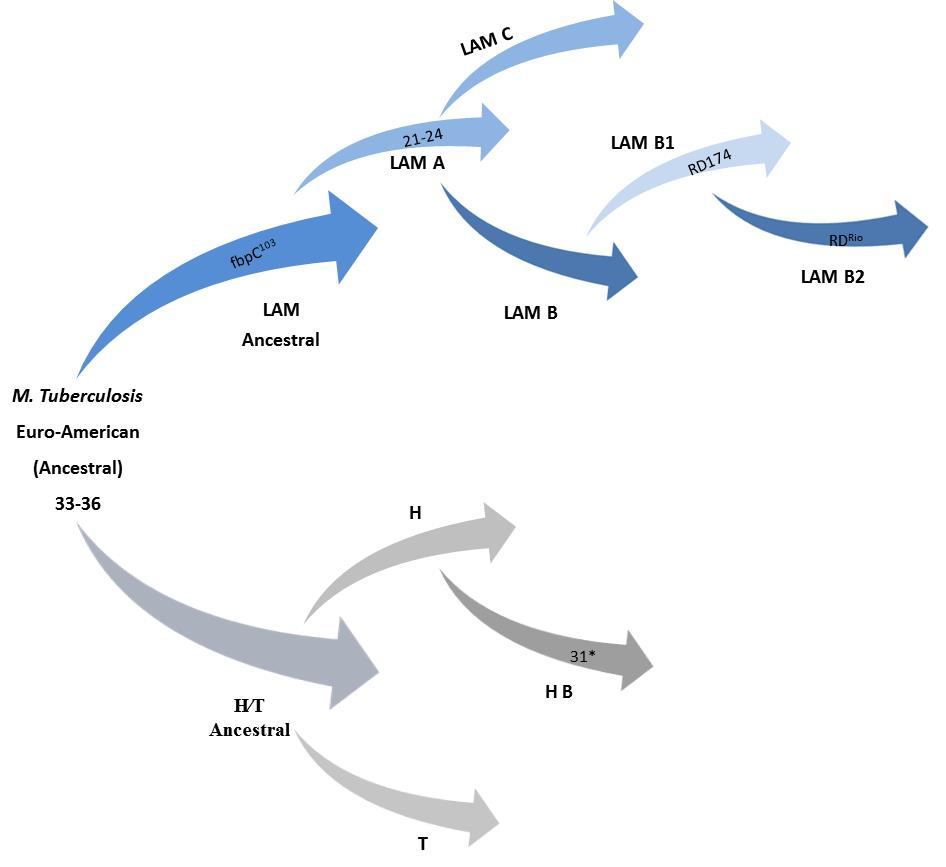
**Figure S3.**

Supplement: Figure S3 — Possible evolution of M. tuberculosis lineage Euro-American (LAM, T and H) according to the markers analyzed in this study. The Euro-American Ancestral evolved into two distinct groups: LAM ancestral and T/H ancestral, characterized by absences of spacers 33–36 and one copy of MIRU24 that is common to all Euro-American lineages. The ancestral LAM strains have fbpC103 and this was the basis of LAM A (LAM9), with additional absence of spacers 21–24, one copy of MTUB30 and two copies of MIRU04 and ETR-A. LAM A on its turn is the basis for two other groups: LAM B (LAM9-LAM4, LAM1-LAM2-LAM5 and LAM6) and LAM C (LAM3). The LAM B evolved from LAM B1, characterized by a deleted RD174 and on its turn to LAM B2, with both deleted RD174 and RDRio. The H/T Ancestral lineage is the origin of both groups H and T (difference only in MIRU-VNTR copies), showing absence of spacers 33–36; additional loss of spacer 31 led to subtype H A, observed in high frequency in this study. (DOC) [file pone.0107747.s003.doc]
